# Ethnic and minority group differences in engagement with COVID-19 vaccination programmes – *at Pandemic Pace; when vaccine confidence in mass rollout meets local vaccine hesitancy*

**DOI:** 10.1186/s13584-021-00467-9

**Published:** 2021-05-27

**Authors:** John A. Reid, Mzwandile A. Mabhala

**Affiliations:** Faculty of Health and Social Care, University of Chester, Riverside Campus, Castle Drive, Chester, CH1 1SL UK

**Keywords:** Vaccine confidence, Vaccine hesitancy, Ethnicity, Inequality, Pandemic pace, Public health workforce

## Abstract

Israel, the UK, the USA, and some other wealthier countries lead in the implementation of COVID-19 vaccine mass vaccination programmes. Evidence from these countries indicates that their ethnic minorities could be as disproportionately disadvantaged in COVID-19 vaccines roll-out as they were affected by COVID-19-related serious illnesses. Their disadvantage is linked to their lower social status and fewer social goods compared with dominant population groups.

Albeit limited by methodology, early studies attribute lower uptake of COVID-19 amongst ethnic minorities to the wider determinants of vaccine uptake, hesitancy or lack of vaccine confidence, including lower levels of trust and greater concerns about vaccine safety. Early sentinel studies are needed in all early adopter countries.

One emerging theme among those of reproductive age in minority communities concerns a worry regarding COVID-19 vaccine’s potential adverse effect on fertility. Respected professional groups reassure this is not a credible rationale. Drug and vaccine regulators use understandable, cautious and conditional language in emergency licencing of new gene-based vaccines. Technical assessments on whether there is any potential genotoxicity or reproductive toxicity should be more emphatic.

From a public health perspective, sentinel studies should identify such community concerns and act early to produce convincing explanations and evidence. Local public health workforces need to be diverse, multiskilled, and able to engage well with minorities and vulnerable groups. The local Directors of Public Health in the UK are based in each local government area and have a remit and opportunity to stimulate speedy action to increase vaccine uptake.

During the rapid Pandemic Pace of the vaccines roll-out, extra efforts to minimise uptake variations are likely to achieve improvements in the next year or two. We expect variations will not disappear however, given that underlying inequalities persist in less inclusive social systems.

## Background

Globally the race is on in rolling out the vaccine against COVID 19. The most successful countries in population vaccination against COVID 19 are Israel, the United Kingdom (UK), the United Arab Emirates (UAE), the United States of America (US) and Chile. The emerging evidence from three of these countries (the UK, US and Israel) indicates that the uptake of the COVID 19 vaccine is lower amongst these countries’ ethnic minorities.

However, the concept of ethnic minorities is often used as though it refers to a constant state of being and homogenous group. In the UK, ethnic minorities refer to all ethnic groups except the White British group, mainly blacks and Arabs and Asians [1]. Israel’s minorities are mainly Arabs, and in the USA the largest groups are African-American, Hispanic/Latino, and Asians with smaller percentages of indigenous populations such as American Indians and Alaskan Natives.

Harris (2021) defines “ethnic minority” as “a group of people who differ in race or colour or national, religious, or cultural origin from the dominant group, often the majority population of the country in which they live [2]“. This definition illustrates that the concept is context-specific; a person could be a member of the minorities in one context and the dominant group in a different context.

It is indisputable that the ethnic minorities in the western context were disproportionately affected by COVID-19-related serious illnesses; it also seems that their engagement with the vaccination programmes is low.

However, the representation of ethnic minorities in the literature and media could erroneously lead to the belief that there is something innate about them that increases their vulnerability and hesitation to engage with covid-19 vaccine programmes. The ethnic minority are socio-demographic and culturally too diverse to share innate qualities that make them vulnerable to COVID-19 or vaccine reluctant or hesitant. The main similarities amongst the ethnic minorities across countries is the social status ascribed to them by the dominant group in each country. Generally, such social status reduces their freedom to fully participate in social goods such as housing, employment, social services, and healthcare services. This creates vulnerability to COVID-19-related serious illnesses, distrust, and lower access and engagement with vaccination programmes.

This commentary is inspired by the study report of respondents to an internet survey reported by Green, Abdullah, Vered and Nitzan in the Israel Journal of Health Policy Research [3]. Like most assessments of the interaction of COVID-19 and ethnic minorities, Green at al; offered several helpful insights into why initial COVID-19 vaccine uptake by Israeli Arab citizens is lower than by the dominant Jewish group [3]. Consistent with most such studies, their initial rapid methodologies restricted it from exploring in-depth how social status ascribed to minorities creates, perpetuates, and sustains health.

This commentary presents our observations of the UK, US and Israel’s experiences in rolling out COVID-19 vaccination, exploring seven underlying themes;

1. Early warning signals that vaccine uptake is lower amongst the minority ethnic groups
2. Determinants of lower uptake by ethnic minorities
3. Need for systematic sentinel alert systems covering variations in uptake of COVID 19 vaccine
4. How minorities feature in COVID-19 vaccine priority frameworks
5. Reproductive health and Infertility as a specific minorities concern
6. Why governments, public health systems, scientists, academics and regulators need to accept a greater share of responsibility around reassurance as more gene-based vaccines become available
7. How the public health workforce can respond effectively to narrow these gaps over time

## Early warning signals that vaccine uptake is low amongst the minority ethnic groups

Countries with strong public health systems have achieved some early successes in rolling out and vaccinating their populations. Some of the early examples of success in rolling out vaccine are Israel, the United Kingdom (UK) and lately, the United States of America (US).

However, the emerging evidence from these countries suggests that ethnic minorities and other vulnerable social groups may be getting further left behind for various reasons, including vaccine hesitancy.

Israel has led worldwide in the fast rollout. Recent analyses clarify factors that influenced Israel’s fast rollout [4, 5]. The first analysis identified 12 factors that enabled Israel’s rapid rollout across the 9.3 million population and attributed Israel’s success to include [4]:

1. pre-existing frameworks for making decisions that involve public health and other experts in vaccine advice;
2. the Israel National Immunization Technical Advisory Group (NITAG);
3. strong systems of electronic population health registers and patient records,
4. high levels of enrolment in healthplan organisations.

Glied pointed to the Israeli vaccine programme’s success arising from the coherent and well-resourced national vaccine strategy, built upon the country’s effective, resilient healthcare system, that is well versed in responding to other emergencies [5].

However, the evidence shows that the vaccine’s uptake was lower amongst the minority groups in Israel [6]. The highest uptake among persons age 50 or older (89%) was among non-Orthodox Jews. The uptake was lower among Israeli Arabs (68%) and Israel’s ultra-Orthodox Jews (62%) [6]. Furthermore, Caspi et al. indicated that in Israel, there was a strong correlation between vaccine acceptance and socioeconomic status with lower uptake amongst those with low socioeconomic status [7]. Based on their research, they recommended that more vaccination promotion effort should be directed to socioeconomically disadvantaged populations [7].

Similar findings have emerged in the earliest major UK studies. One of the largest early UK retrospective cohort studies; covered an overall 961,580 vaccinated people within 23.4 million GP registered study population [8]. Their analysis showed ‘substantial ethnic divergence in the uptake of vaccine amongst the over 80 age group living outside care homes’ [8]. It also shows, albeit to a lesser magnitude, the effects of deprivation indicators. The proportion vaccinated to date was highest among white people (42.6%), with South Asian 29.5%, and lowest among black people at 20.5% [8]. Sixteen more detailed ethnic categories were analysed, and black ethnicity categories had low rates generally, while Bangladeshi/British Bangladeshi (23.0%) and Pakistani/British Pakistani (22.8%) categories also had the lowest uptake rates. This is of great public health concern given that the over age 80, Black and Minority Ethnic (BAME) groups have highest COVID-19 morbidity/mortality risks [8]. Further evidence reinforcing such concerns came from varied sources including government statistics [9], the Royal College of General Practitioners [10], the Royal Society, the British Academy [11], and the Royal Society of Public Health [12].

Health service providers’ concerns were also being raised about lower uptake in NHS healthcare workers from different BAME backgrounds [13]. Since the healthcare workers were likely to have equal or better access to vaccines, the lower uptake appeared to be related primarily to vaccine hesitancy.

## Determinants of lower uptake by ethnic minorities

The UK Scientific Advisory Group for Emergencies (SAGE) ethnicity sub-group review in December 2020 looked at factors influencing COVID-19 vaccine uptake among minority ethnic groups and other minority issues [14, 15]. Their work was set against historical lower ethnic minority uptake in other vaccine programmes, including those introduced last decade, such as against Rotavirus in children and Shingles (VZ) in population over age 70 years [14, 15]. Their reviews identified four barriers to vaccine uptake:

- Lower trust and confidence in vaccine efficacy and safety
- Lower perception of risk
- Inconvenience and access barriers, including costs
- Context and socio-demographic variation, including levels of education.

The study by Green and colleagues challenges the theory that lower perception of infection risk is the main factor in COVID-19 lower uptake [3]. In their study, the apparent higher educated and younger Arab females have higher levels of reluctance. They highlighted that there was a mismatch with apparently greater confidence and good uptake in standard vaccine programmes for children and families by educated Arabs. They confirm that, in general, the uptake of the COVID-19 vaccine is higher amongst socially advantaged groups regardless of ethnicity [3]. Previous studies from South Africa, where Blacks are the majority, appear to confirm the vital proposition that a group’s socioeconomic status within society is more the determinant for vaccine uptake than ethnicity [16]. This study found that vaccine uptake among deprived black South Africans was significantly lower than the least deprived [16].

## Need for systematic sentinel alert systems covering variations in uptake of COVID 19 vaccines

Timely information about the progress of rollout can guide policymakers and program directors at all levels. And, of course, sentinel information should be tailored to local demographics.

In Israel, an immediate challenge was to address the hesitancy among the educated women in the Arab population [3]. In the UK, the Office for National Statistics reports on variations in vaccination uptake, including minorities’ concerns about side effects, safety, and possible long-term effects [17].

In the US, the earliest report of variation in uptake of COVID 19 amongst the minorities came from CDC, but had had a large percentage of missing data on ethnicity or race [18]. The Kaiser Family Foundation’s (KFF) COVID-19 vaccine dashboard recording early responses in each State to questions eliciting vaccine hesitancy or lack of ‘vaccine enthusiasm’ [19]. Concerns emerging in minority groups include [19]:

- That vaccines may contain the live (coronavirus) virus
- That there will be out of pocket expenses, that vaccine is not free and might require health coverage documentation, or that personal data collected could be transferred to other agencies,
- That younger age groups are more hesitant than older and more likely to wait and see (how events develop, such as a single-dose vaccine)
- Short-term fears of losing time from work to longer-term concerns about Infertility.
- That health professionals are held in the highest regard and with reasonably high regard for public health agencies. Also, that family, friends, and religious leaders are relatively higher key influencers in a sizeable proportion and need to be positively engaged.

Early insights should allow policymakers and program directors to address such minority group concerns about composition of vaccines, extra personal costs or loss of income from more severe side effects. Vaccination programmes are mainstream social programmes that everybody is expected to participate in. Arguably, in societies where minorities are relegated towards society’s margins, they may be more reluctant to participate in those mainstream programmes. Early reports, cited above, could not attribute underlying causation. Studies on perception and opinion offer partial insight but do not report on structural facilitators or barriers to service use. There was little initial reportage on wider determinants of vaccine uptake in relation to social position, power, and inclusiveness.

## How minorities feature in COVID-19 vaccine priority frameworks

This pandemic has demonstrated both the importance of developing a prioritised set of vaccine recommendations and recognising that the recommendations and their priority may need to change over time. In the UK, the Joint Committee for Vaccination and Immunisation (JCVI) initially prioritised vaccine recommendations based on clinical risk as determined by age, clinical conditions, and health and social care worker status [20]. In their decision about a list of priorities they considered ethical issues and evidence - specifically relating to inequalities and ethnic minorities [21], but concluded on balance to proceed as above. As the pandemic progresses, adjustments are being made on priorities, such as the inclusion of homeless people and people in detention settings in March 2021 [22].

Although the initial JCVI recommendations were based on the above evidence, the pandemic has demonstrated that other factors should also be considered. These include an ‘intersectional human rights’ approach balancing risk and benefits across society [23]. Therefore, the vaccination programmes should balance preserving scientific integrity and independence along with ethical frameworks such as informed consenting adults and building wider trust and engagement with minorities. Such debates may be helped by comparative analysis with recent decisions in other countries, such as the European Centre for Disease Prevention and Control (ECDC) assessments of NITAGs and country priorities in EU/EEA states [24]. Broader principles, such as the legitimacy principle for vaccine prioritisation advocated by WHO, could be adopted from the beginning, through the earliest consideration of the long list of potentially vulnerable socio-demographic groups that they identified [25].

## Reproductive health and infertility as a specific minorities concern

Green and colleagues’ study shows that educated Arab women respondents raised some concerns about the potential effects of the COVID-19 vaccine on fertility. This was important for them since they were, on average, about ten years younger than Jewish respondents [3].

The UK based Association of Reproductive and Clinical Scientists and the British Fertility Society, the Human Fertilisation and Embryo and Authority, and the Royal College of Obstetrics and Gynaecology, have issued statements to counteract the misinformation that COVID-19 vaccines could potentially impact male or female fertility [26–28].

The British Islamic Medical Association (BIMA) also provided reassuring information for patients in regard to infertility concerns, and in BIMA’s case, also on other myths such as about COVID-19 vaccine containing non-Halal or alcohol-based constituents [29].

There has been measured and cautionary reassurance around vaccine safety, as there is ongoing research to establish with certainty the safety of these vaccines. However, the modern scientific movement towards Gene-Based Vaccine technology deserves further consideration.

## Why governments, public health systems, scientists, academics and regulators need to accept a greater share of responsibility around reassurance as more gene-based-vaccines become available

Vaccine hesitancy has proven to be a global concern. Addressing it will require each country sponsoring a vaccination program to address this issue for its own population. Since some lessons about addressing vaccine hesitancy are likely to be generally applicable, ideally countries would work together to understand and spread the best practice [30]. Countries all have some degree of vaccine mistrust and will need various study methods to explore their own communities’ concerns and the underlying reasons. For instance, a recent analysis of COVID-19 anti-vaccination social media content in Poland highlighted ‘angrier’ versus ‘happier’ arguments and comments [31]. Concerns expressed around morality, religion, ideology and personal testimonies about children/others harmed were angrier, along with comments involving civil liberties, conspiracy/search for truth theories, and safety and effectiveness concerns.

There is a need to understand better if some rapid COVID-19 vaccine policy and technical reviews have built or eroded public trust or vaccine confidence. It has been contended that India’s government may have been overly hasty with lack of transparency in India’s vaccine (Covaxin) licensing arrangements [32].

Normally vaccine licensing relies on the publication of satisfactory Phase 3 human studies. It appears that in some countries, such as India and Russia, rollout followed smaller Phase 2 studies [33–36]. There are concerns about the potential over-closeness of vaccine regulators and public health decision-makers to their national governments that may have influenced vaccine policy.

The continuing metanalysis of studies into COVID-19 during pregnancy shows that women being of non-white ethnic origin might also be a risk factor for severe covid-19 infection [37]. This is a strong argument for pre-conceptual vaccination rather than vaccination in pregnancy.

There are large numbers of Gene-Based-Vaccine candidates under development for COVID-19 [38]. These advancing technologies are open to mistrust by anti-science and anti-vaccine advocates. Regulatory and expert bodies need to take a joined-up approach to communicate the best evidence to speedily counter vaccine non-confidence narratives, as with infertility concerns.

Any ensuing controversy or disagreement among medical, public health and international scientific communities is a likely media trigger that can help spread uncertainty worldwide.

All countries must have modern public health risk communication strategies. In the UK, public health risk communication advice was first issued in 1997. It was generated by communication mistakes and harsh lessons from public health crises of confidence and controversies, such as with the variant Creutzfeldt-Jacob (vCJD) outbreak from Bovine Spongiform Encephalopathy (BSE) or ‘mad-cow disease’ infecting beef, Salmonella in UK egg supplies, and the Mumps, Measles and Rubella (MMR) vaccine controversy [39]. Bennett, Calman and co-authors, updated their thinking in 2010 on the social amplification of risk, and fright factors and media triggers [40]. Six of their eleven fright factors apply to COVID-19 vaccines, such as genetically engineered vaccines and worries about fertility. Fright factors include concerns about perceived threats to health from unfamiliar sources or that may pose some long-term danger to pregnant women or future generations or are subject to contradictory statements from responsible sources.

Several agencies and expert groups are engaged in with COVID-19 vaccine rollout within the UK, such as the JCVI and MRHA (Medicines and Healthcare Products Regulatory Agency). All perform meaningful and valued roles but may give scope for inconsistency in the message or in emphasis. The UK COVID-19 Vaccine Task Force has led forcefully on vaccine procurement, supporting vaccine industry development, and supporting international aid and supplies. However, their 2020 end of year report made claims for a comprehensive communication campaign [41]. This is partly accurate but does not fully acknowledge the challenges in successfully engaging minority groups. Further detailed guidance from the UK Race Disparities Unit encourages targeted local action and engagement with support from community champions and other local leadership [42].

A wider communications challenge is the understandably cautious technical and legal language used in conditional approvals or emergency-use licensing [43–47]. COVID-19 infection places its viral RNA in abundance within our bodies’ cells. Yet, public hesitancy over using vaccines to place small segments of this virus’s RNA in our cells.

Medicines and vaccines regulators should inspire wider trust and take credit for rapid comprehensive reviews of vaccine trial studies. However, overly enthusiastic rhetoric of leaping forward and a revolution in vaccine technology [48] might reinforce some reluctance. A measured look-back at lessons from recent Gene-Based-Vaccine uses in Ebola outbreaks emphasises taking all communities with you from the start, from experiences with affected indigenous African communities [49].

## How the public health workforce can respond effectively to narrow these gaps over time

Rigorous approaches are needed to set up and run successful vaccination programmes [50]. Many different vaccination services needed to be sited across large populations in England [51]. Mass COVID-19 vaccine clinic venues may not suit some ethnic/cultural or faith-based minority groups where privacy is harder to maintain, where language difficulties could arise, and the pace of vaccination leaves little room for meaningful dialogue at that point, given each vaccine clinical encounter usually takes only a few minutes. We do not know yet if some UK venues (e.g. racecourses) or smaller church-related (Christian) venues have influenced ethnic minorities’ choices. However, some faith-based and ethnic group communities are now more actively involved in local and more tailored communications in the UK. There are efforts to locate vaccination clinics in more accepted local assets, such as worship places, including mosques. A variety of minority language materials are now available. Local COVID-19 vaccine community champions and influencers in minority groups are being identified and encouraged.

A Kaiser Family Foundation recently reported many local public health and state’s innovative and responsive measures scattered across the USA. All responses should be evaluated rigorously to learn for future vaccine rollout-out and wider public health practice to reduce health inequalities [52]. Their vaccine COVID-19 dashboard gathers useful lessons from the USA. It represents a coordinated well-resourced approach by a non-statutory research body that actively seeks out and reports data on public perception and State-level systems responses early on and by tracking it over time [52].

The 1997 UK public health risk communication guidance advised to engage and build trust carefully, not to overlook basic stakeholder concerns, and to investigate and communicate in a two-way process throughout the timescales [37]. Razai and colleagues more recently advised on the ‘need to engage, listen with respect, communicate effectively, and offer practical support to those who have yet to make up their minds about the vaccine’ [53]. England’s local public health leadership falls to the Directors of Public Health (DsPH), who moved from the NHS into local government systems in 2013. There is hope that local Directors of Public Health will help in vaccine catch-up for local minorities, and a light touch national review will shortly look at the range of local activities after enquiring with DsPH.

Firstly, the pivotal role of DsPH has been better recognised recently, rather than being marginalised by central government earlier in the pandemic. Secondly, DsPH are geographically closer to their populations (e.g. about 1 DPH per 320,000 in North-West England’s region of about 7.3 million people, closer than in Israel where regional public health Directors cover populations over 1 m people). Thirdly there is some diversity of gender and ethnicity in that group of 23 DsPH. Finally, the UK multidisciplinary specialist public health workforce allows DsPH to represent differing skillsets. There may need to be a greater focus on cultural competency skills in the whole public health workforce in our future curricula and training programmes. Useful tools and materials have been collated for healthcare professionals from Public Health England [54] and the NHS Race and Health Observatory [55]. Guidance from UK SAGE [14] includes ongoing community engagement, tailored communication shared by trusted sources, and avoiding stigmatisation and discrimination.

## Conclusions - pandemic pace – the race for better vaccines continues along with increasing levels of population vaccination. How far can countries protect such vulnerable minority groups in the next year or two?

The race for vaccines goes on with the early pacesetters showing urgent minority group issues to addressed. Ultimately this is also a race between humanity versus the virus, as it spreads and mutates. For public health professionals, our race is between advancing our societies’ organised efforts to promote widest population protection and equity to achieve universal coverage while battling the brakes of community hesitancy and systems disruption.

Pandemic pace implies accelerated emergency development of biotechnologies, with an understanding that ‘every day counts’ [56]. Perceptions are prone to change in communities and need tracking. Distrust, however, propagated, may take various forms around the world. In the USA, there is a high current distrust of vaccine among male Republican Party voters [57]. This is not a minority ethnic grouping but still needs to be understood, for instance as to whether motivated by political positioning, or anti-science or male-associated perceptions. In a recently published French study conducted last summer, there is higher mistrust of vaccines made in China, and, worryingly, 40 (32.2%) out of the 124 healthcare workers responding would make ‘outright refusal’ [58] In Australia, opinion has moved, between August 2020 to January 2021, away from vaccine confidence, notably in females, and in Indigenous Australians and those who speak a language other than English in their own homes [59].

The rollout of vaccines against SARS-CoV-2 in several countries have had differential uptake between the majority and minority populations. Although there has been some evidence of vaccine hesitancy and resistance in all populations within each country, the phenomenon appears to be greater among the minority populations. This is particularly unfortunate since these same minority populations have had higher attack rates of disease due to SARS-CoV-2 and should benefit more from the vaccines. It looks possible for localised public health systems to identify and diminish vaccine hesitancy through local disease and risk-perception surveillance and by further building trust and even closer engagement with ethnic and other minorities and by adapting vaccination services for greater fit with each minority community.

Public health policy measures from the seven themes above should include:

1. All countries should develop strong vaccine data systems, to analyse and report uptake data suitable to their minorities composition. Ethnicity terminologies in our countries can be contentious and global thinking about minorities classification need to be updated [60].
2. Public health research should focus on the underlying determinants of disadvantage and exclusion as well as vaccine perceptions and vaccine access factors. They should avoid furthering stigma, blame and further discrimination for excluded groups and report objectively on structural determinants and any discriminatory or racist policies or exclusion.
3. Vaccine programs should promote speedy sentinel alerting studies, such as with the Israel example [3] and the others above shown from USA, UK and other countries. These should complement responsive routine vaccine surveillance systems.
4. All country-level vaccine prioritising frameworks should recognise higher infection risk in minorities and specify strategic goals for high uptake and equity. Where vaccine certification or passports are introduced [61], they should show whether they hinder or promote uptake by minorities. We also recognise, but do not comment here, that major vaccine access equity problems for Palestinian populations need to be addressed fully in Gaza and West Bank.
5. Public health professions and scientists should give coordinated attention to any concerns about perceptions of potential long-term effects, such as about reproductive toxicity and Infertility. A danger is with newer gene-based vaccines, including genetically modified technologies for other target infections, that wider vaccine distrust could grow, giving greater energy to modern infodemics or to sources of active health misinformation and anti-vaccination proponents.
6. Policymakers should work closely with regulatory agencies to evaluate both efficacy and safety and pursue rapid, open scientific publication of evidence reviews. The pandemic’s emergency vaccine authorisations have understandable conditionality and cautious language. Uncertainty or precautionary messages can be exploited by those with anti-vaccination motives and encourage greater ‘hesitancy’ in some social groups. Policymakers should update frameworks for assessing vaccine efficacy and effectiveness further than recently proposed [62], to embrace quantitative and qualitative analysis of vaccine perceptions at local community and minority group level. Inevitably, as pandemic vaccines are rolled out fast to millions of people, there will be rare clinical conditions reported that might be linked to vaccines, such as clotting disorders [63]. These have the potential to fuel vaccine hesitancy. However, if policymakers assess and handle these well, they could minimise hesitancy. They may pause or suspend vaccine use meanwhile for some of the population or all. Countries may vary in their historical precautionary stances on vaccines. Ultimately, vaccination costs and benefits should always be assessed and discussed openly.
7. Public health professional systems and institutions will need to be reviewed with regard to community engagement. The public health workforces will probably need to be strengthened in capacity and capability to identify and respond better to communicable disease inequalities and variations in vaccine uptake at the locality level and integrate enhanced supportive expertise from academic, clinical and other professional experts. We must work closely with local GP and other primary care colleagues who are trusted in their communities and should be supported in their community-based commitments and innovations [64].

It is not too late for policymakers to ensure that health inequalities are not further exacerbated and to learn lessons from early mass vaccination programmes [65]. Already in the UK some of these hesitancy gaps are lessening [66]. However, vaccine safety concerns about rarer disorders are to be expected as newer vaccines appear and large populations are immunised. Strong engagement with minority groups should continue to build trust. However, greater attention should be given to the fundamental social and structural determinants of the distrust of governments and state institutions. Otherwise it is unlikely that the gaps in uptake will disappear altogether.

## Data Availability

Cited articles references supplied.

## Abbreviations

BAME: Black and Ethnic Minority
JCVI: (UK) Joint Committee for Vaccination and Immunisation
NITAG: National Immunization Technical Advisory Group
SAGE: (United Kingdom) Scientific Advisory Group for Emergencies
